# Morphological Differences between Larvae of the *Ciona intestinalis* Species Complex: Hints for a Valid Taxonomic Definition of Distinct Species

**DOI:** 10.1371/journal.pone.0122879

**Published:** 2015-05-08

**Authors:** Roberta Pennati, Gentile Francesco Ficetola, Riccardo Brunetti, Federico Caicci, Fabio Gasparini, Francesca Griggio, Atsuko Sato, Thomas Stach, Sabrina Kaul-Strehlow, Carmela Gissi, Lucia Manni

**Affiliations:** 1 Dipartimento di Bioscienze, Università degli Studi di Milano, Milano, Italy; 2 Dipartimento di Scienze dell’Ambiente e del Territorio e di Scienze della Terra, Università di Milano Bicocca, Milano, Italy; 3 Laboratoire d’Ecologie Alpine (LECA), Université Grenoble-Alpes, Grenoble, France; 4 Museo di Storia Naturale, Venezia, Italy; 5 Dipartimento di Biologia, Università degli Studi di Padova, Padova, Italy; 6 Department of Zoology, University of Oxford, Oxford, United Kingdom; 7 Humboldt-Universität zu Berlin, Institut fur Lebenswissenschaften, Vergleichende Zoologie, Berlin, Germany; 8 Department of Integrative Zoology, University of Vienna, Vienna, Austria; Consiglio Nazionale delle Ricerche (CNR), ITALY

## Abstract

The cosmopolitan ascidian *Ciona intestinalis* is the most common model species of Tunicata, the sister-group of Vertebrata, and widely used in developmental biology, genomics and evolutionary studies. Recently, molecular studies suggested the presence of cryptic species hidden within the *C*. *intestinalis* species, namely *C*. *intestinalis* type A and type B. So far, no substantial morphological differences have been identified between individuals belonging to the two types. Here we present morphometric, immunohistochemical, and histological analyses, as well as 3-D reconstructions, of late larvae obtained by cross-fertilization experiments of molecularly determined type A and type B adults, sampled in different seasons and in four different localities. Our data point to quantitative and qualitative differences in the trunk shape of larvae belonging to the two types. In particular, type B larvae exhibit a longer pre-oral lobe, longer and relatively narrower total body length, and a shorter ocellus-tail distance than type A larvae. All these differences were found to be statistically significant in a Discriminant Analysis. Depending on the number of analyzed parameters, the obtained discriminant function was able to correctly classify > 93% of the larvae, with the remaining misclassified larvae attributable to the existence of intra-type seasonal variability. No larval differences were observed at the level of histology and immunohistochemical localization of peripheral sensory neurons. We conclude that type A and type B are two distinct species that can be distinguished on the basis of larval morphology and molecular data. Since the identified larval differences appear to be valid diagnostic characters, we suggest to raise both types to the rank of species and to assign them distinct names.

## Introduction

*Ciona intestinalis* is an ascidian firstly described by Linnaeus in Northern European Seas (the so called “*Oceano europaeo*” of *Sist*. *Nat*., 1789, Gmelin edition, pag. 3123) and then recorded in all oceans, from high to low latitudes. The original and very short description of this species was extended by Roule [1] and subsequently by Millar [2]. Remarkably, in spite of the high number of papers describing specimens collected in different localities, no author found substantial intra-specific differences concerning the adult morphological and taxonomically-valid characters reported in Millar’s description [2].

In the last decades, *C*. *intestinalis* has become a model invertebrate chordate in various fields of biology, from developmental biology to evo-devo and comparative genomics [3]. The publication in 2002 of the nuclear genome draft of an individual sampled in California gave further impulse to the study of this model organism and helped to clarify the evolutionary origin of chordate novelties [4]. As a model, *C*. *intestinalis* offers several advantages, because it combines the chordate body plan with the handiness of an invertebrate that produces thousands of fast developing embryos, easily reared in seawater.

The cosmopolitan distribution of *C*. *intestinalis* and the ease of sampling in nature represent practical advantages for the collection of these animals for scientific purposes. For example, several research centers around the world offer collection and shipping services of wild-type animals to the research community (e.g., the Station Biologique de Roscoff, the Stazione Zoologica Anton Dohrn of Naples, and the Japanese NBRP project [5]). Thus, the research community works in different regions of the globe with individuals of different origin and having different genetic background. Obviously, this *modus operandi* is based on the assumption that all natural populations belong to the same species. However, in the last years this assumption was challenged by several genetic and molecular analyses. These studies ultimately indicated the existence of a *C*. *intestinalis* species complex, including at least four distinct “taxa”, genetically highly divergent and easily distinguishable on a molecular basis [6,7,8,9,10]. Depending on the author, these taxa were described as distinct “types” or “species”: here we will adopt the term “types”, because it is neutral from a systematic and taxonomical point of view. Going into details, type C and type D appear to be restricted to the Mediterranean and Black Sea, respectively [10], while type A and type B have disjoint global distributions and seem to be highly invasive [6,7,9,10]. Type A was found in the Mediterranean Sea, the Pacific Ocean (Australia, Japan, New Zealand, South Korea and West coast of North America), and the Atlantic coast of South Africa. Type B was found in the European and Canadian coasts of the North Atlantic Ocean [6,7,9], as well as in the Bohai and Yellow Seas (China) [10]. Types A and B coexist only in the English Channel and in some localities of the French Atlantic coast (e.g., in Plymouth, UK, and Brest, France), which then are sympatric areas where hybridization and introgression phenomena have been partially examined [6,8,11].

The sequence divergence between type A and type B was initially noticed by the comparison of large nuclear genome portions of a British and two Pacific specimens [9]. After this pioneer study, the geographical distribution of these two types and their genetic divergence were more deeply investigated using a number of molecular markers: the *cox1* barcode sequence, microsatellites, structural features of the mitochondrial genome, and several nuclear loci characterized by sequencing or restriction analyses [7,10,12,13,14]. In all these studies, the phylogenetic reconstructions placed type A and type B individuals in two distant and well-supported monophyletic clades, and agreed on the conclusion that the identified level of sequence divergence is surprisingly high for, and therefore incompatible with the expected intra-species variability. Thus, from the genetic point of view, type A and type B should be considered as cryptic species.

A further element of the puzzle of the existence of cryptic species within *C*. *intestinalis* was provided by the examination of hybridization and introgression between the two types [13,14,15] and by a recent genome-wide population genetics study [11]. Through the analysis of 852 protein-coding loci, Roux and collaborators [11] came to the conclusion that type A and type B originated by a speciation event that occurred about 3.8 Mya. After that, the two gene pools remained in complete isolation for > 3 million years, allowing the accumulation of numerous genetic incompatibilities throughout the genomes. Then, about 15,000 years ago, there was a recent secondary contact between type A and type B, with gene introgression occurring at a relatively low rate and mostly unidirectionally [11]. This complex and highly dynamic scenario of the diversification of type A and type B is probably one of the causes for the difficulties in the clear identification and delimitation of distinct species in the *C*. *intestinalis* species complex. Indeed, to be considered separate species, populations must have been separated long enough to evolve unique derived diagnostic characters.

According to the biological species concept, investigations on reproductive isolation are fundamental for the demonstration of the existence of distinct species. In this respect, few studies examined the efficiency of fertilization and the viability/fertility of the F1 offspring in hetero-type crosses of *C*. *intestinalis* (type A eggs x type B sperm and *vice versa*). Surprisingly, these studies reported different results ranging from the presence of at least some compatibility barriers up to the existence of reproductively isolated entities within the *C*. *intestinalis* species complex. The observed wide variability in the results of the hybridization experiments seems to depend on the allopatric/sympatric nature and the geographic origin of the parents, as well as on the different experimental conditions of the fertilization assays (i.e., developmental temperature, salinity, etc.).

In spite of the wealth of molecular data, only two studies compared the morphology of *C*. *intestinalis* type A *versus* type B, and in both cases only the anatomy of the adult was taken into account [6,14]. In particular, Caputi and collaborators [6] found that the two *C*. *intestinalis* types can be distinguished by the spermiduct pigmentation: type A individuals have red or bright orange genital papillae and an uncolored duct, while type B shows pigmentation only in the duct. Later on, Sato *et al* [14] proposed three inherited morphological characters as useful markers to distinguish types A and B in the field (*i*.*e*., by eye and without help of optical instruments): (1) body color; (2) presence/absence of tubercles on the siphons; (3) yellow/orange pigments at the distal end of siphons. Unfortunately, these characters were not present in all individuals of a given type, so that the definitive assignment of an individual to type A or type B had to be confirmed by molecular analyses. The ambiguous distribution of these morphological characters hampered a valid description of type A and type B as distinct species, according to the morphological species concept, and consequently also prevented the assignment of distinct names based on the International Code of Zoological Nomenclature.

In this study, we focus our attention on the *C*. *intestinalis* larval morphology, and look at diagnostic characters able to discriminate type A from type B larvae. The study of hybrid larvae is not addressed here, because it necessitates standardization and optimization of the hybridization procedure depending on the examined parental individuals. Since *C*. *intestinalis* is widely used as model in developmental biology, its larval development has been already extensively investigated even in absence of complete information on the type/parental origin. These developmental studies often include the publication of images of whole-mount preparations (based on *in situ* hybridization, immunohistochemical staining, or fluorescence labelling) of the larvae, that could provide useful data for the comparison of type A *versus* type B morphology. Thus, identifying morphological larval differences is of great practical importance. Therefore, we here present morphometric and histological analyses, as well as 3D reconstructions of type A and type B larvae obtained by adults sampled in different seasons and in different localities. Our analyses identify diagnostic characters permitting to clearly distinguish late larvae belonging to the two types. Besides, these data strengthen the results we obtained in a parallel study on the morphological re-description of adult individuals belonging to type A and type B [16]. Overall, we conclude that type A and type B are indeed two distinct species that can be distinguished not only from the genetic but also from the morphological point of view.

## Results and Discussion

### Morphometric analyses of type A and type B larvae

*C*. *intestinalis*, like almost all tunicates, is an organism with an indirect development producing tadpole larvae. These larvae have a prominent trunk, where the majority of the organs is located, and a locomotory tail. Larvae possess transient structures that will be lost during metamorphosis (*e*.*g*., the outer cuticular layer and the outer compartment of the tunic, the adhesive organ with the three papillae, the sensory vesicle containing the ocellus and the otolith, the visceral ganglion and the nerves), and structures that are prospective juvenile organs. Among these latter structures, there is the pre-oral lobe, a wide anterior body-cavity in the anterior part of the trunk, comprised between the pharynx and the anterior epidermis. At metamorphosis, the anterior epidermis is destined to thicken into a disc of ectoderm for juvenile adhesion to the substratum, whereas the pre-oral lobe will elongate in the stalk sustaining the animal body [17,18].

**Fig 1:**
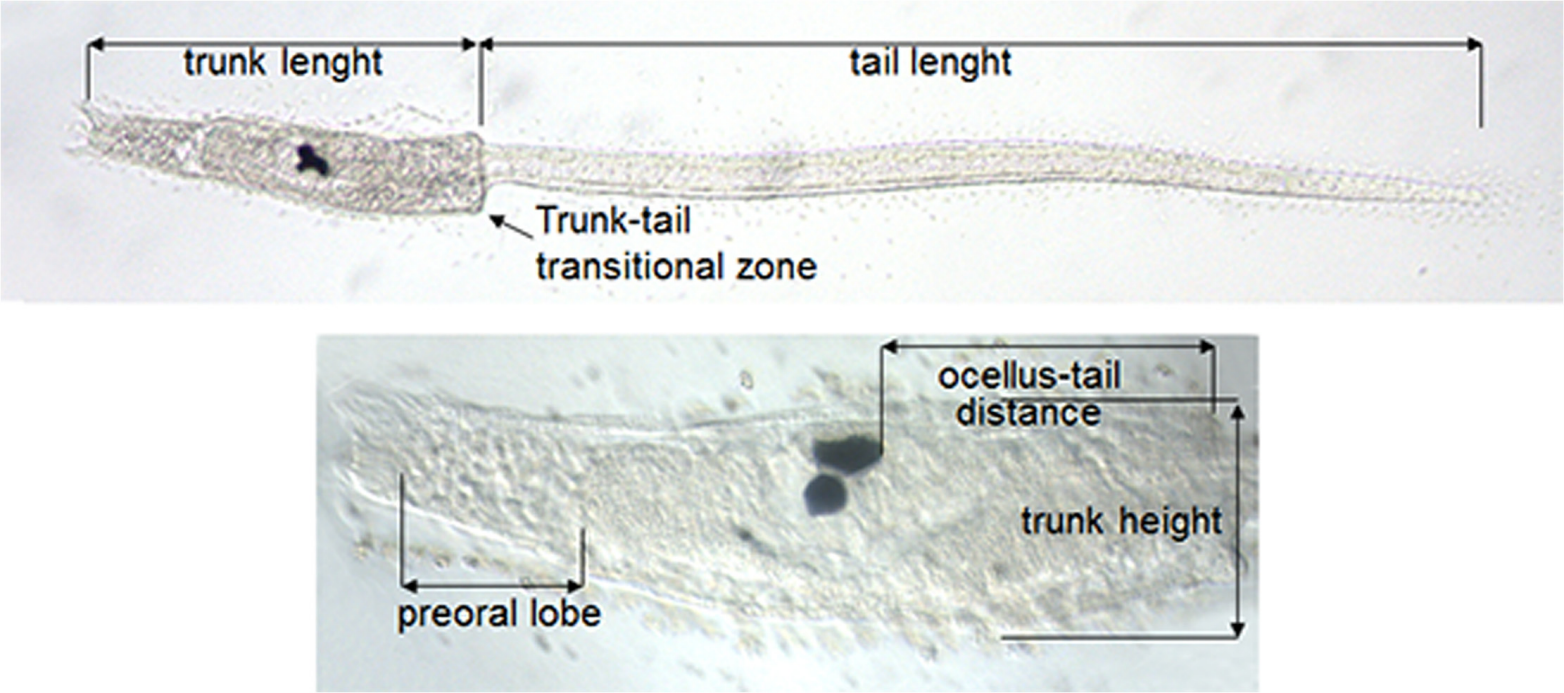
Morphometric measurements performed on late-swimming larvae of *Ciona intestinalis*.

Considering the overall structure and development of the larvae, our morphometric analyses concerned the five parameters described in Fig 1, *i*.*e*., length of the tail; length of the trunk; maximum height of the trunk; length of the pre-oral lobe; distance between the ocellus and the insertion point of the tail.

All analyses were carried out on late-swimming larva 24 h post fertilization at 18°C, corresponding to stage 29 according to the FABA2 database (http://chordate.bpni.bio.keio.ac.jp/faba2/2.2/top.html) and stage 2 according to Chiba [19]. This stage was selected because at this step the larvae have completed their development, which consists mainly in a process of elongation occurring after hatching.

Overall, we analysed 260 type A and 166 type B larvae derived from 18 homo-type crosses of individuals sampled in four distinct localities (Plymouth and Roscoff for type B; Plymouth, Venice and Naples for type A) and in two seasons (Table 1).

**Table 1:** Homo-type crosses and resulting larvae analysed in this study.

**Crosses**	**Source**	**# larvae**	**# crosses**	**Date**	**Dataset**
AxA	Venice	96	4	2013, Spring	
AxA	Naples	40	1	2013, Spring	
AxA	Plymouth	90 ^a^	3	2011, Autumn	SV, MA, CD
AxA	Plymouth	34	2	2011, Spring	SV, MA, CD
BxB	Roscoff	48	1	2013, Spring	SV
BxB	Roscoff	44 ^b^	2	2012, Autumn	SV
BxB	Roscoff	17	1	2011, Autumn	SV
BxB	Plymouth	27	2	2011, Spring	SV, MA, CD
BxB	Plymouth	30 ^c^	2	2011, Autumn	SV, MA, CD

SV: dataset used for seasonal variability investigations; MA: larvae and parents for which the type attribution was based on molecular analyses; CD: dataset analyzed for investigation of the character displacement phenomenon.

a: including 15 larvae without measurement of the pre-oral lobe length.

b: including 19 larvae without measurement of the pre-oral lobe length.

c: including 12 larvae without measurement of the pre-oral lobe length and one without measurement of the ocellus-tail distance.

**Fig 2:**
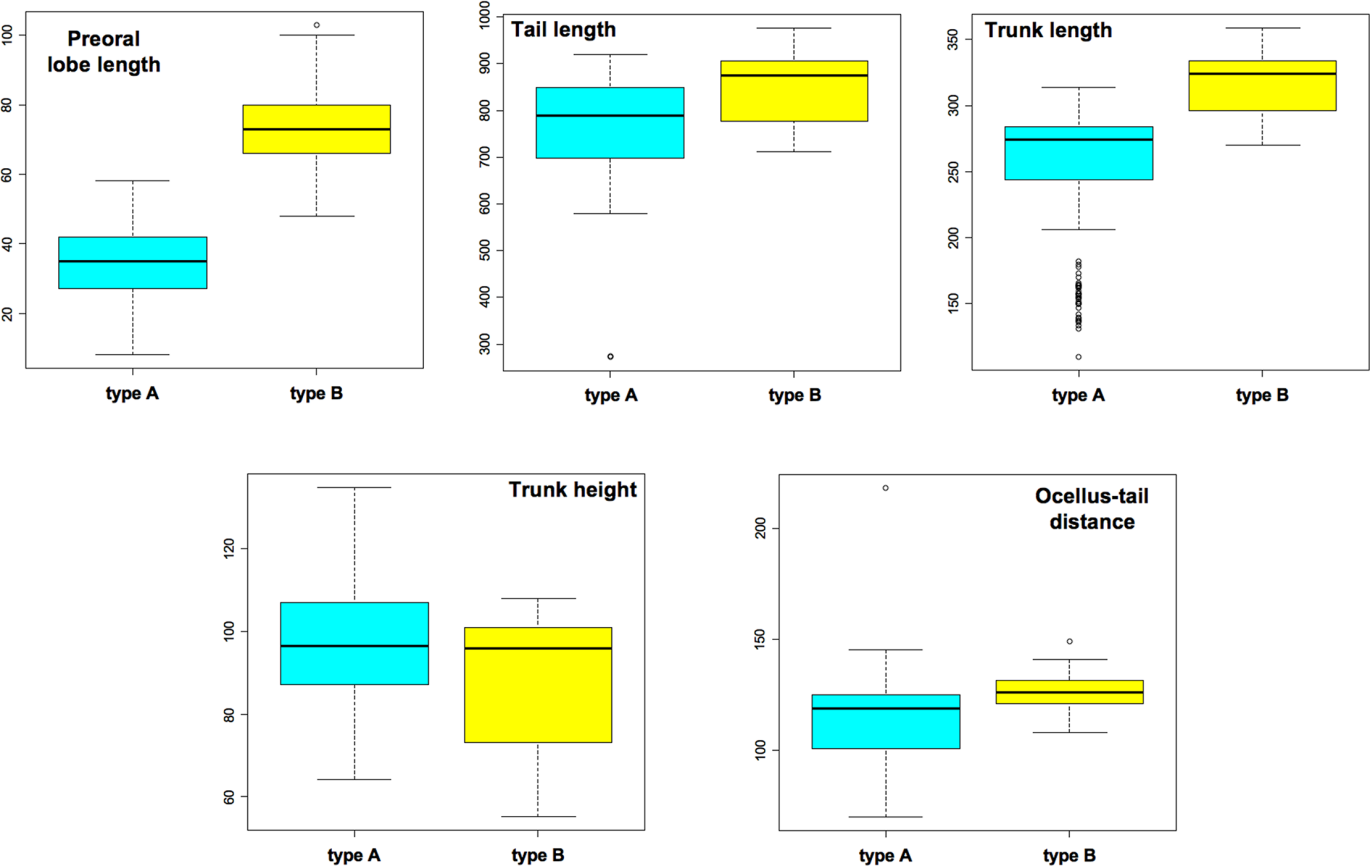
Box plots of the five analyzed larval parameters. Only larvae derived from the spring crosses listed in Table 1 have been considered. Measurements are reported in μm.

Statistical analyses were based on linear mixed models and performed on the dataset including only larvae obtained in spring (Table 1). Statistically significant differences between type A and type B were detected in all analyzed morphological parameters, except for the maximum height of the trunk (Fig 2 and Table 2). Differences were particularly strong for the other parameters representing features of the trunk (p< 0.001).

**Table 2:** Results of mixed models testing differences between type A and type B larvae for the five analyzed morphological parameters.

**Dependent variable**	***F***	**d.f.**	***P***
Preoral lobe length	113.4	1,4	<0.001
Tail length	14.5	1,4	0.019
Trunk length	158.5	1,4	<0.001
Trunk max. height	1.4	1,4	0.307
Ocellus-tail distance	134.7	1,4	<0.001

All models included study site and clutch identity as random factors.

**Fig 3:**
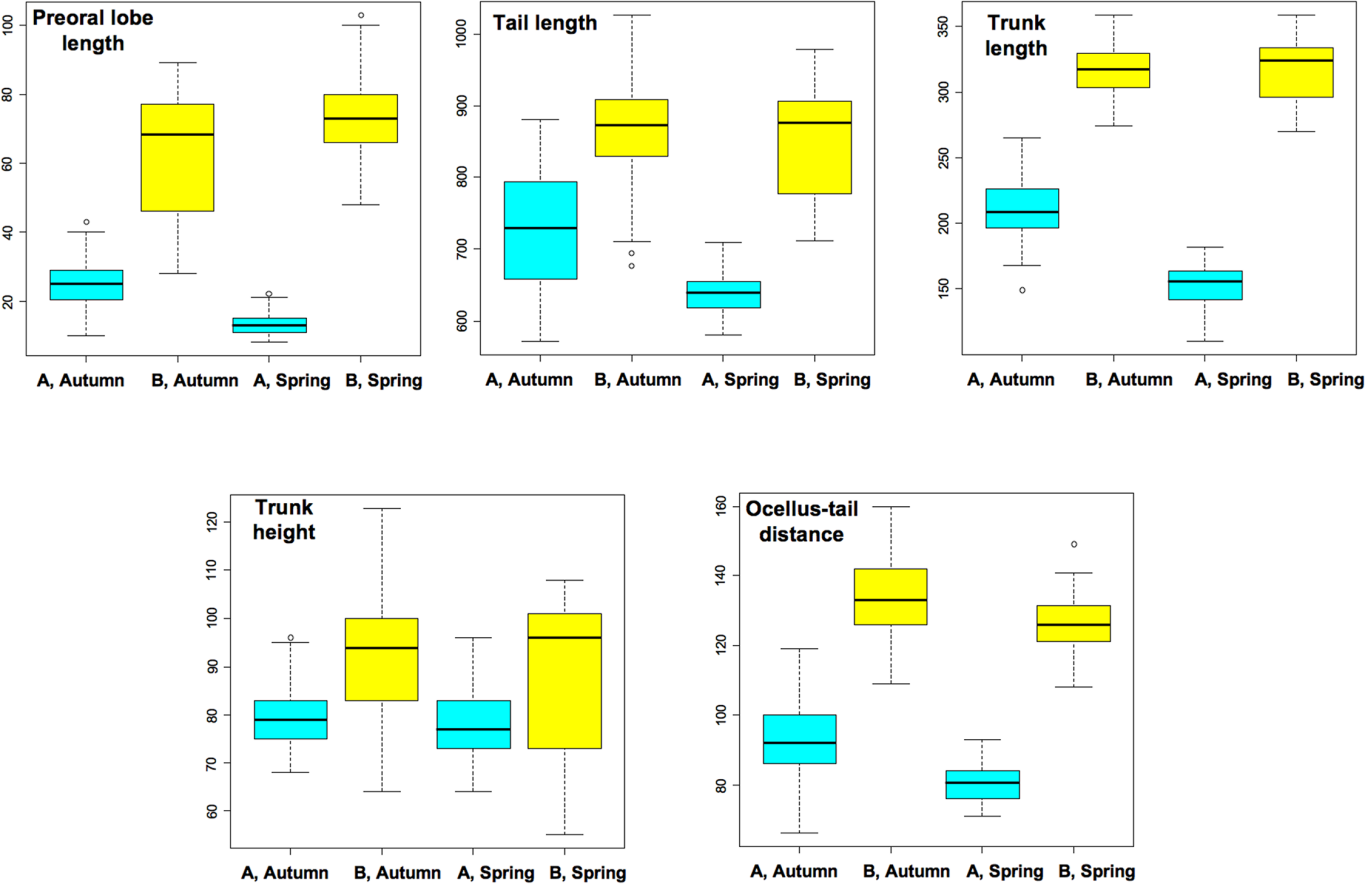
Morphometric data of larvae obtained from adults collected in Plymouth and Roscoff in two different seasons. The analyzed dataset is indicated as SV in Table 1. Type A larvae are indicated in sky blue, type B larvae in yellow. Symbols: *, p<0.05; **, p<0.01; ***, p<0.001.

In order to verify the possible existence of larval size variability due to seasonal effects, we carried out further investigations only on the larvae obtained from individuals sampled in the same locality both in spring and autumn (dataset seasonal variability, SV, in Table 1). The box plots of the five analyzed parameters and the corresponding statistical analysis based on linear mixed models are reported in Fig 3 and Table 3. These analyses confirmed the existence of significant differences between type A and type B sampled in the same season for all five investigated parameters (see the “Species” column in Table 3). Moreover, these analyses also revealed the existence of an intra-type seasonal variability that is statistically significant for all parameters except for the pre-oral lobe length (see the “Season” column of Table 3). For trunk length and for the pre-oral lobe length, the differences between species were particularly strong in spring, as shown by statistically significant interactions (Table 3).

**Table 3:** Results of mixed models testing differences between type A and type B larvae for five morphological parameters between seasons.

**Dependent variables**	**Independent variables**								
	**Species**			**Season**			**Species × season**		
	*F*	d.f.	*P*	*F*	d.f.	*P*	*F*	d.f.	*P*
Pre-oral lobe length	127.5	1, 28	**<0.001**	0.1	1, 28	0.715	21.8	1, 28	**<0.001**
Tail length	87.7	1, 28	**<0.001**	9.0	1, 28	**0.006**	0.9	1, 28	0.343
Trunk length	425.1	1, 28	**<0.001**	36.1	1, 28	**<0.001**	16.2	1, 28	**<0.001**
Trunk max. height	11.9	1, 28	**0.002**	7.1	1, 28	**0.013**	0.7	1, 28	0.407
Ocellus-tail distance	281.1	1, 28	**<0.001**	24.4	1, 28	**<0.001**	0.4	1, 28	0.527

All models included study site and clutch identity as random factors.

**Fig 4:**
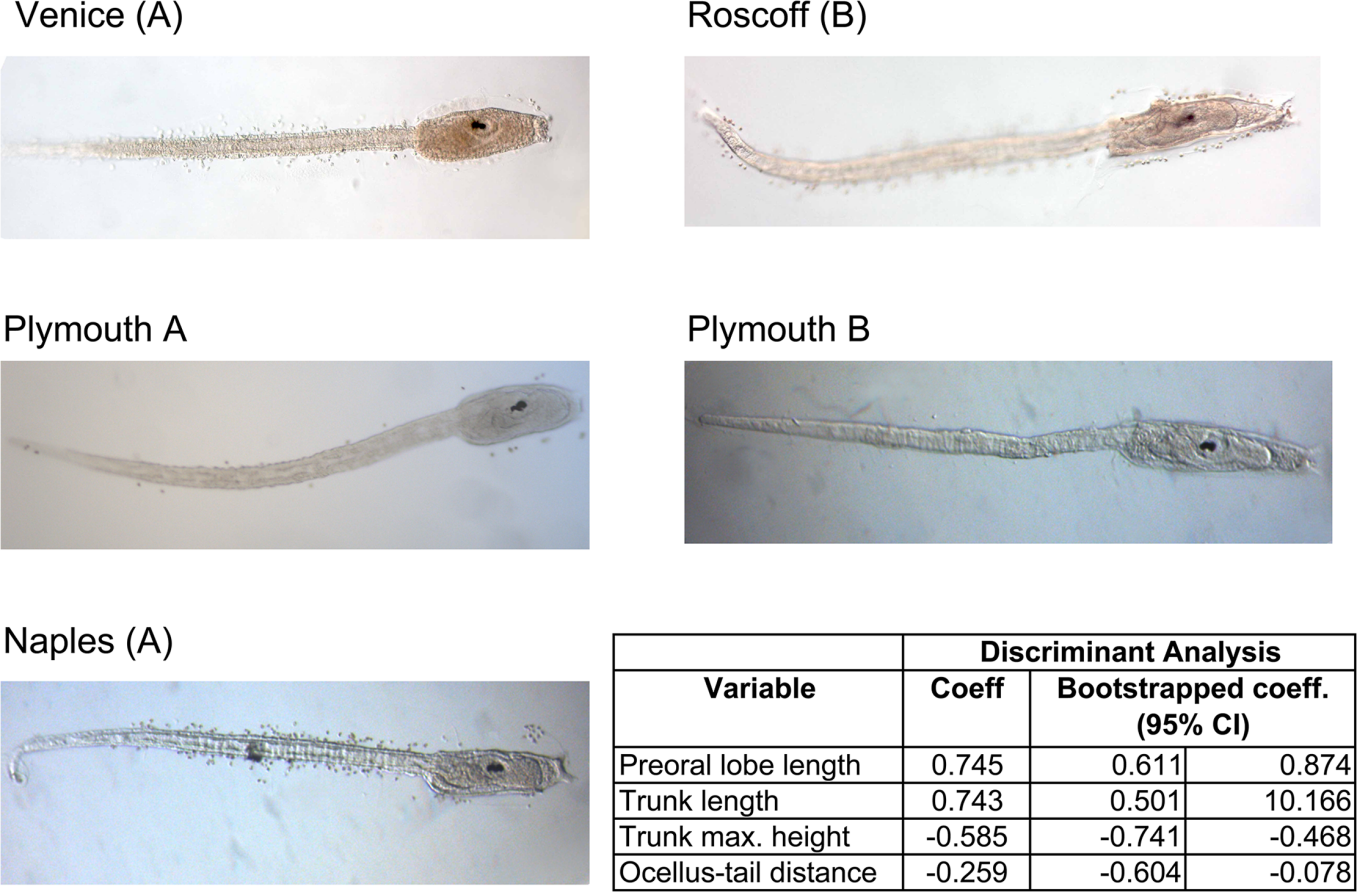
Representative pictures of type A and type B larvae coming from different localities, and results of the Discriminant Analyses (DA). The analyses were carried out on the whole larval dataset of Table 1. Symbols: *, p<0.05; **, p<0.01; ***, p<0.001.

To ascertain whether morphological data could be used to unequivocally determine the type identity, we carried out a Discriminant Analysis (DA) on the whole dataset of Table 1. As shown in Fig 4, type B is clearly identified by having longer pre-oral lobe, longer and relatively narrower total body length, and shorter ocellus-tail distance than type A. Stepwise selection of discriminant function identified four variables maximizing the probability of correct classification of individuals: pre-oral lobe length, trunk length, trunk height and ocellus-tail distance. The discriminant function (named “4v”) based on these four variables strictly related to the trunk, so without tail length, was able to correctly classify 95.5% of larvae. This discriminant function was highly significant (Wilk’s Λ = 0.277, χ^2^_4_ = 481.79, *P* < 0.001). Moreover, the discriminant analysis was still able to correctly classify most of the larvae, even if the number of analysed parameters was reduced. In particular, using the ratio between pre-oral lobe length and trunk height (PL/TH ratio) as the sole variable, the new cross-validated discriminant function (named “1v”) was statistically significant (Wilk’s Λ = 0.372, χ^2^_1_ = 373.05, *P* < 0.001) and correctly classified 93.2% of larvae. The two discriminant equations able to classify larvae on the basis of both (a) the four selected variables and (b) the single PL/TH variable are reported in Supporting S1 File. The inability of these discriminant functions to correctly classify the 100% of the analyzed larvae could be explained by the previously observed intra-species seasonal variability, which is statistically significant for almost all considered parameters (see “Season” columns in Table 3). So, we anticipate that a better sampling of the larvae, taking more widely into account the morphometric seasonal variability, could allow setting up discriminant function(s) with a better resolving power, possibly usable as a hands-on tool for distinguishing type A from type B larvae.

In addition to the above-described quantitative differences, we observed that the trunk profile in the larval transitional region between trunk and tail (described in Fig 1) is squared in type B larvae, while it is rounded in type A larvae (compare larvae of the two types in Fig 4). Although almost all type B larvae can be distinguished from type A using this character, it is a qualitative feature quite difficult to be evaluated objectively, *i*.*e*., without bias, so it was not included in our statistical analyses.

In conclusion, our analyses revealed that larvae of the two types strongly differ in trunk shape at the stage of late-swimming larvae. This difference can be easily observed at the stereomicroscope, also on living specimens, and can be used to almost unequivocally discriminate the two types of larvae. Thus, even in absence of molecular data or of unambiguous information on the parent sampling location, morphometric analyses on the larva can allow its assignment to a given *C*. *intestinalis* type.

Interestingly, larval differences between type A and type B were particularly strong in the Plymouth populations, *i*.*e*., in the sole known locality where the two types live in sympatry. This is evident from the result of a discriminant analysis carried only on the Plymouth populations (CD dataset of Table 1): in this case the four parameters strictly related to the trunk (excluding the tail length) correctly classified 99.3% of individuals, with only one misclassified individual. This situation recalls a classic case of ecological character displacement, which stems from resource competition [20,21]. Although deep ecological studies will be necessary to ascertain the ecological character displacement, we emphasize that some criteria necessary to determine it are already indicated by our data. In particular, we refer to the fact that the greater morphological divergence of sympatric larvae is here statistical proved.

In order to verify the validity and the practical applicability of the larval morphometric approach to discriminate type A from type B specimens, we performed morphometric analyses on 52 photographies of larvae of *C*. *intestinalis* selected from a number of developmental biology publications (S2 File). Due to the absence of precise data on the larval stage in almost all papers, we analyzed only larvae that appeared very similar to late-swimming larvae. Moreover, due to the lack of molecular data, the information on the locality of animal sampling were used to ascertain if these larvae derived from type A or type B adults. On the whole, we collected 45 larval photos of type A and 7 of type B. For each photo, we measured as many as possible of the five above-described morphometric parameters, and then, according to the available measurements, we applied one or both the discriminant equations (the 4v and 1v equations reported in S1 File) to classify these larvae. As specified in S2 File, only in three cases we observed differences in the type classification derived from the application of both the discrimination functions. Forty-four of the total 45 analyzed type A larvae were correctly classified by one or both the discriminant equations, while only one of the seven type B larvae was correctly classified. Remarkably, the only type A larva classified as type B was sampled on the Atlantic coasts of NW Spain (Ria de Vigo [22]), *i*.*e*., in a Spanish locality where molecular screening of *C*. *intestinalis* was never carried out. Therefore, also considering that Vigo is on the Atlantic coast, the presence of type B individuals in this locality cannot be excluded. Thus, this larva could be a type B specimen. Although the obtained morphometric classification did not work perfectly for type B, we consider this approach very promising and easily improvable through the definition of discriminant function(s) based on a larger larval sample. Indeed, the incorrect classifications that we obtained can be explained by several elements, such as the existence of intra-specific seasonal variability in the larval morphology; the analysis of photographies of larvae corresponding to a mix of stages rather than only to the late-swimming larval stage (on which we defined the discriminant functions); the examination of many different *C*. *intestinalis* populations sampled around the world. On the basis of these considerations, we conclude that the larval morphometry is an effective and very promising approach for *C*. *intestinalis* type discrimination.

### Histology of type A and type B larvae

**Fig 5:**
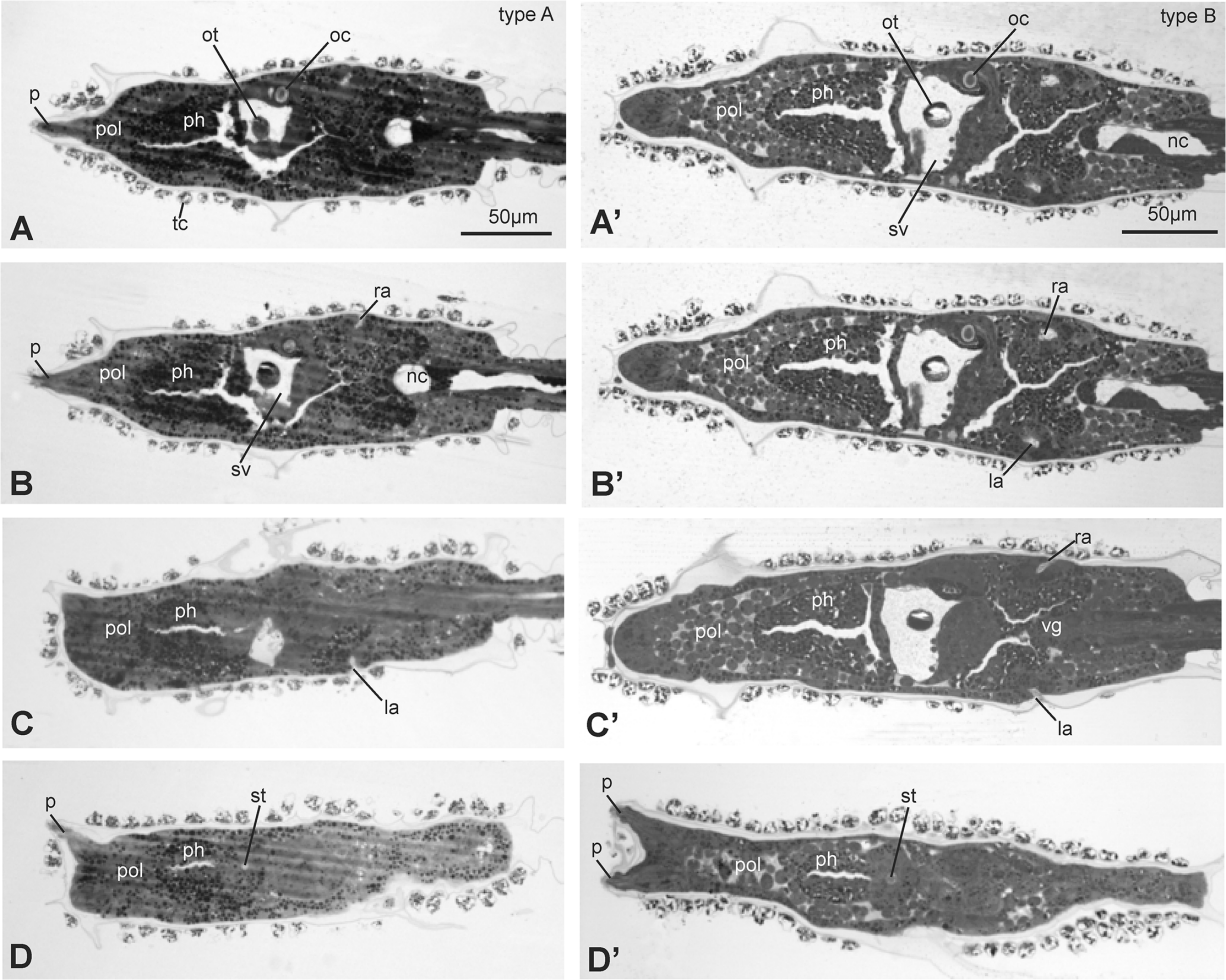
Frontal sections, from ventral (A and A’) to dorsal (D and D’) side of late larvae of type A (A-D) and type B (A’-D’). The sections were selected from two complete series of sections and show the relationships among stomodeum, pharynx, nervous system and atria. Toluidine blue. la, left atrium; nc, notochord; oc, ocellus; ot, otolith; p, papilla; ph, pharynx; pol, pre-oral lobe; ra, right atrium; st, stomodeum; sv, sensory vesicle; tc, test cell; vg, visceral ganglion. Scale bars A and A’: 50 μm; all figures have the same enlargement.

**Fig 6:**
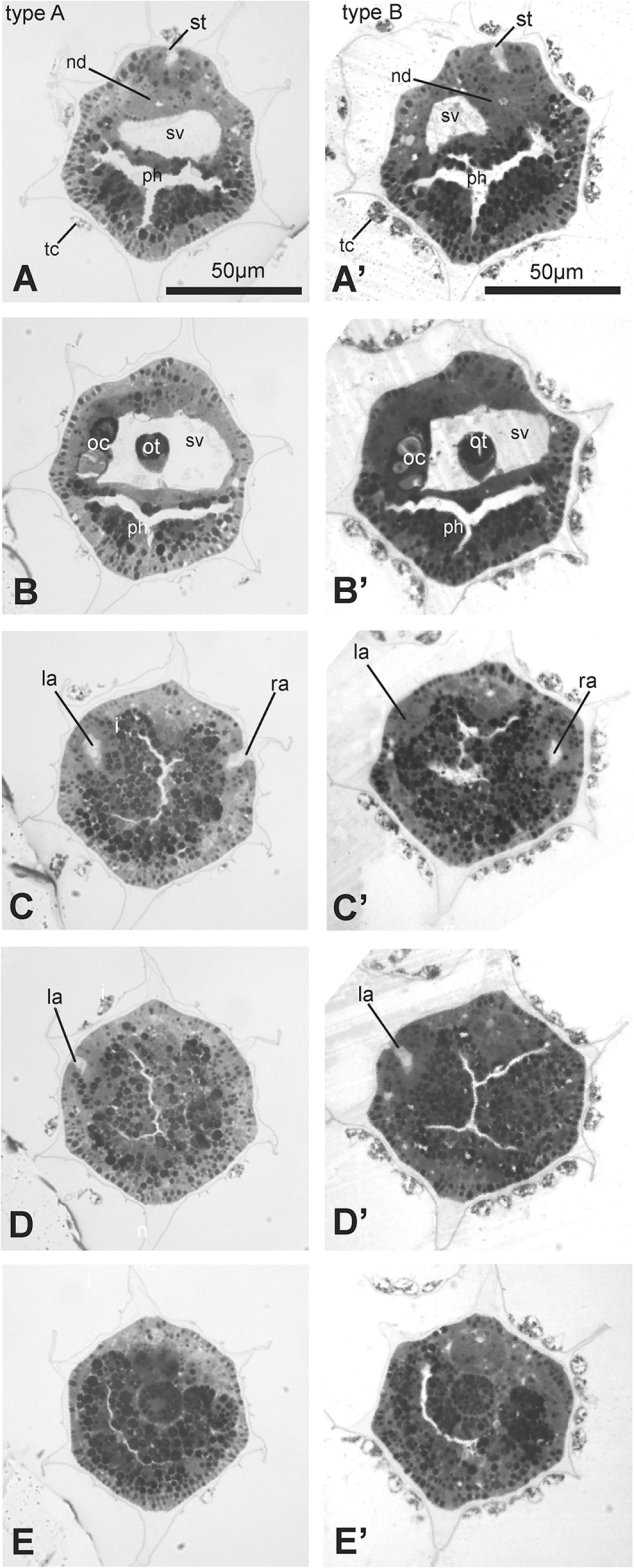
Transverse sections, from anterior (A and A’) to posterior (E and E’) of late larvae of type A (A-E) and type B (A’-E’). The sections were selected from two complete series of sections and show the relationships among stomodeum, pharynx, nervous system and atria. Toluidine blue. I, intestine; la, left atrium; n, nerve cord; nc, notochord; nd, neurohypophyseal duct; oc, ocellus; ot, otolith; ph, pharynx; ra, right atrium; st, stomodeum; sv, sensory vesicle; tc, test cell. Scale bars A and A’: 50 μm; all figures have same enlargement.

In order to find more subtle differences between type A and type B larvae, we analyzed by light microscopy the histology of three different larval stages: early, mid and late (0,1, 2 according to Chiba *et al* [19], also corresponding to stage 27, 28, 29 according to the FABA2 database http://chordate.bpni.bio.keio.ac.jp/faba2/2.2/top.html). We collected complete series of cross, frontal and sagittal sections of trunk and part of the tail for early, mid and late larvae originating in Venice (type A) and Roscoff (type B). The analyses were performed on three larvae for each stage and for each geographical source and type. For a direct evaluation of possible differences between larvae of the two types, we chose to compare sections showing some reference organs. In particular, we analyzed sections at the level of the oral siphon rudiment (*i.e.*, the stomodeum), the otolith, the paired atria (*i.e.*, the rudiments of the adult atrial siphon) and the anterior end of the notochord (at the posterior trunk level) (Figs 5 and 6).

In general, larvae of the same type were uniform in terms of dimension and anatomy. An overview of sections showed that the larvae of the two types shared the same cytological properties, such as cell dimension, tissue consistency, quality of tissue staining, and yolk content (Figs 5 and 6). Moreover, the overall histology of type A and type B larvae was in agreement with previously published data on *C*. *intestinalis* [23]. In particular, the reciprocal positions of stomodeum, sensory vesicle, atria and notochord appeared the same in the two types.

However, our histological data better highlighted the significant differences between type A and type B already observed through morphometric analyses: 1) larvae of type B were longer and wider than larvae of type A; 2) the pre-oral lobe was clearly longer and wider in type B larvae than in type A. This emerged clearly by the comparison of the juxtaposed frontal sections shown in Fig 5. These differences were evident in all the three analyzed larval stages, although they were more pronounced in late than in early and mid larvae.

**Fig 7:**
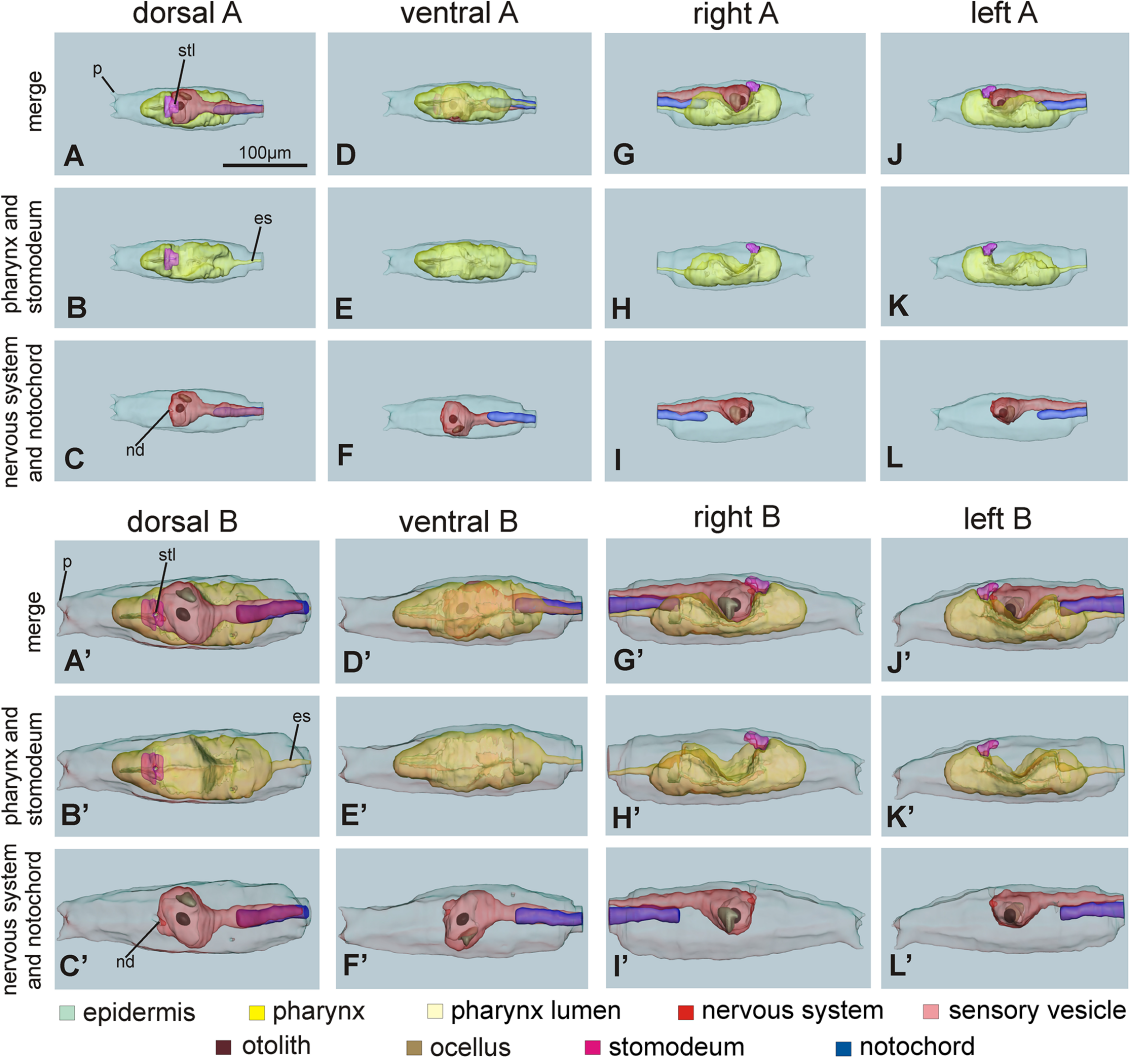
3D reconstructions of whole larvae of type A (A-L) and type B (A’-L’). The reconstructions are viewed from dorsal (A-C, and A’-C’), ventral (D-F and D’-F’), right (G-I and G’-I’) and left (J-L and J’-L’) sides. (See also the 3D-PDF of S3 and S4 Files; requires Acrobat Reader version 7.0 or higher). Reconstructions A-A’, D-D’, G-G’ an J-J’ represent the main larval structures: the epidermis (green), the stomodeum (shocking pink), the pharynx (yellow) with its lumen (pale yellow), the nervous system (red), the sensory vesicle lumen (pink) with the otolith (brown) and the ocellus (pale brown) and the notochord (blue). Reconstructions B-B’, E-E’, H-H’ and K-K’ show only the epidermis, the pharynx (with its lumen) and the stomodeum; reconstructions C-C’, F-F’, I-I’ and L-L’ show only the epidermis, the complete nervous system and the notochord. es: endodermal strand; nd: neurohypophyseal duct lumen; p: papilla; stl: stomodeum lumen. All pictures have the same enlargement (see the scale bar in A).

**Fig 8:**
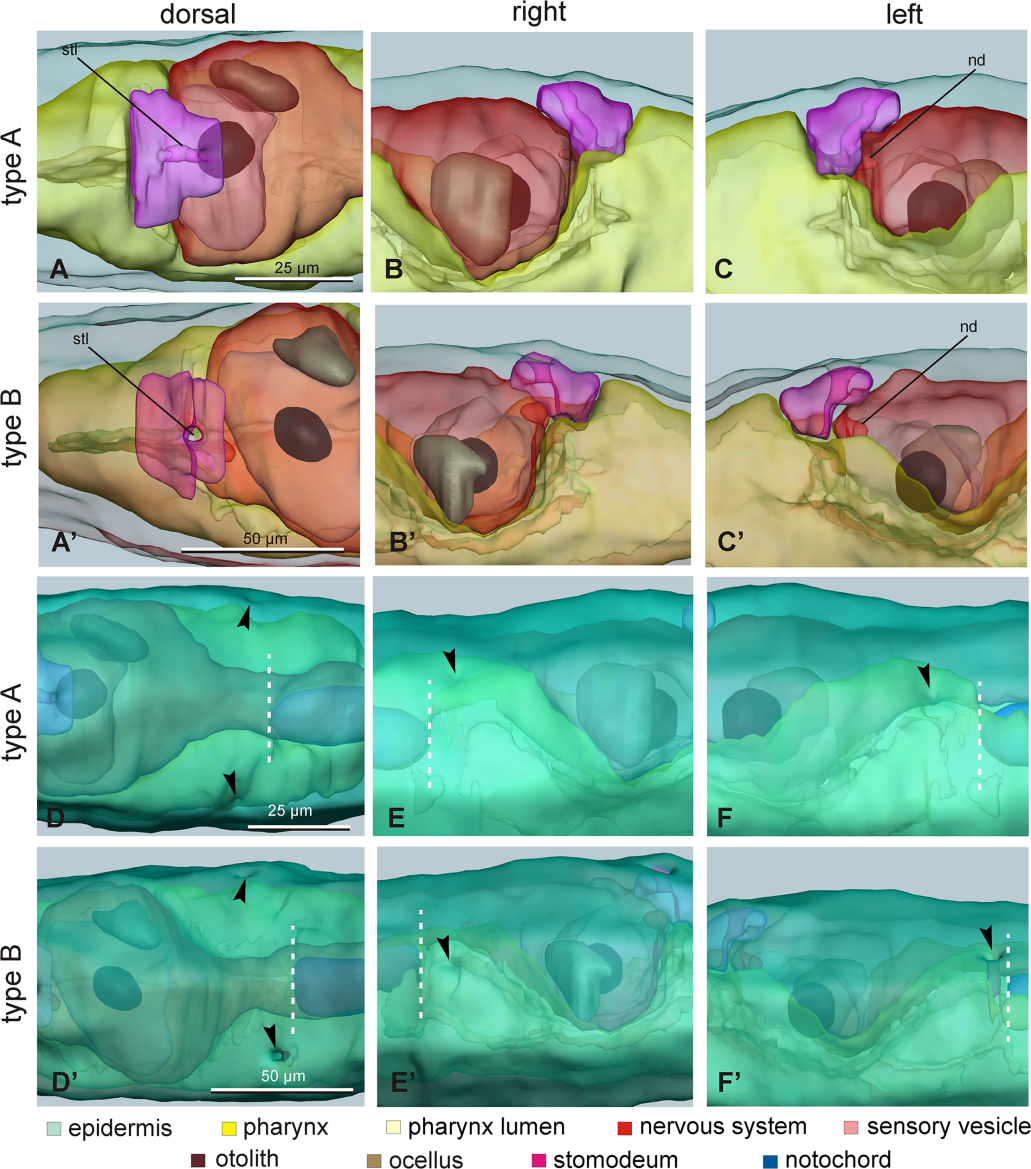
Details of 3D reconstructions of larvae. A-C’. Detail of the stomodeum (shocking pink) and its relationships with the pharynx (yellow) and the neurohypophyseal duct in larvae of type A (A-C) and type B (A’-C’), viewed from dorsal (A, A’), right (B, B’) and left (C, C’) sides. nd: neurohypophyseal duct; stl: stomodeum lumen; green: epidermis, pale yellow: pharynx lumen, red: nervous system, pink: sensory vesicle lumen; brown: otolith; pale brown: ocellus. D-F’. Detail of posterior region of cephalenteron to compare the position of the two atria (arrowheads) and the anterior end of the notochord (white dotted line) in larvae of type A (D-F) and type B (D’-F’), viewed from dorsal (D, D’), right (E, E’) and left (F, F’) sides. The epidermis (green) has been intensely colored to allow visualization of atria; internal structures are semi-hidden. Enlargements are the same in each row of pictures (see the scale bars in the first column).

Figs 7 and 8 show the 3D reconstruction of the late larval stage for one representative type A individual and one representative type B individual, respectively. Three-dimensional animations of larvae of the two types can be accessed in S3 and S4 Files. These representative larvae were selected for their typical morphology from a pool of late larvae embedded in resin. For each larva, the 3D reconstruction was based on 1μm thick serial cross sections of a single specimen (276 sections for the type A larva and 233 sections for the type B larva). These reconstructions extensively represented the above-mentioned differences between type A and type B larvae, and also revealed other minor differences. The meaning of these minor differences is presently ambiguous and should be confirmed analyzing a larger sample size: at the moment, we cannot state whether these differences are structural differences between the two types or reflect an intra-type variability imputable to specificities of the two selected specimens. Among these differences, we emphasize that the lumen of the stomodeum was vertical in the type B larva, whereas it bended forward in the type A larva; moreover, the notochord tip was closer to the atria in type B than in type A (Fig 8).

### Peripheral sensory neurons in type A and type B larvae

**Fig 9:**
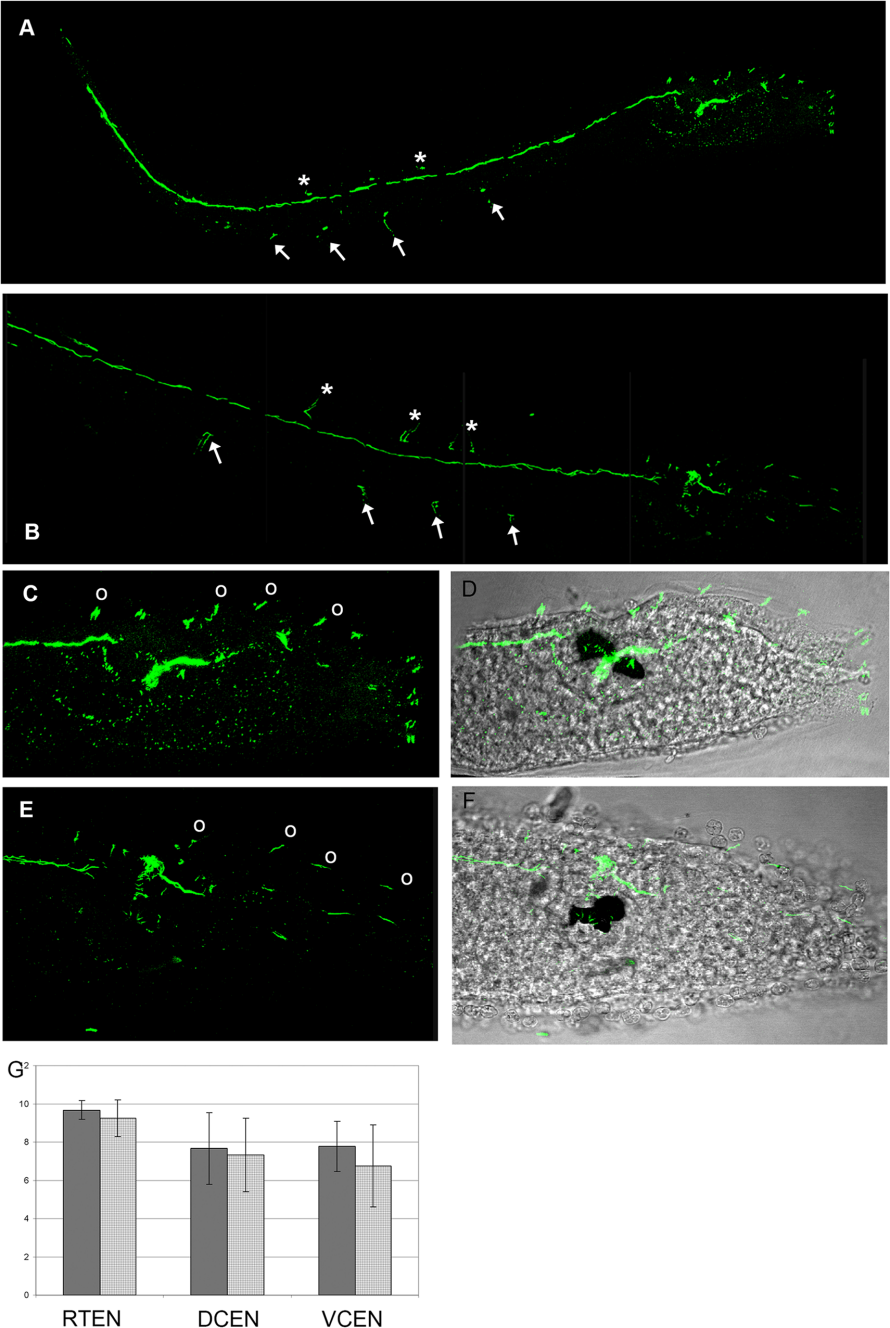
Immunolabeling of nervous fibres in late larvae of type A (A,C,D) and type B (B,E,F). A,B confocal laser microscope imagines of whole larvae. Asterisk indicate dorsal caudal epidermal neurons, arrows indicate ventral caudal epidermal neurons. C,E. Magnifications of the trunk regions. Circles indicate trunk epidermal neurons. D,F. Superimposition of C and D with transmission microscope images. G. Graph showing the mean number of neurons ± standard deviation. DCEN: dorsal caudal epidermal neurons of the tail; VCEN ventral caudal epidermal neurons of the tail; TEN: trunk epidermal neurons.

As further quantitative larval characters, we compared the number of trunk epidermal sensory neurons between late-swimming larvae of type A (9 samples from 3 homo-type crosses of Venice individuals) and type B (12 samples from 3 homo-type crosses of Roscoff individuals). Possible differences in these neurons could indeed indicate the existence of differences in the peripheral nervous system organization between the two *C*. *intestinalis* types. Epidermal neurons were identified by immunostaining with an anti-acetylated tubulin antibody (see Materials and Methods) and subdivided into dorsal caudal epidermal neurons (DCEN) of the tail, ventral caudal epidermal neurons (VCEN) of the tail, and trunk epidermal neurons (TEN) (Fig 9A–9F) [24]. Statistical analysis showed that there is not significant difference in the number of each neuron category between larvae belonging to the two types (U-test of Wilcoxon: TEN: W = 67.5, P = 0.313; VCEN: W = 75, P = 0.1355; DCEN: W = 59, P = 0.7455) (Fig 9G).

We also carefully analyzed sensory neurons in the papillae, i.e., in the three conical projections of the anterior tip of the trunk, two dorsal and one ventral. In *C*. *intestinalis*, papillae were described as simple, non-eversible, and constituted of secreting cells, axial columnar cells, primary sensory neurons, and undifferentiated ectodermal cells [25]. At the onset of metamorphosis, the papillae play an important role in substrate selection, and serve as attachment devices by secretion of sticky substances [17,26]. Since papillae can be useful for ascidian species identification (see, for example, [27], and the following paragraph), we compared the papillae of late larvae between types A and type B. We performed both a light (using three samples for type) and an immunofluorescence (using 10 samples for type) microscopy analysis. No evident histological difference was found between the papillae of type A and B larvae (data not shown): in both types, the papillae exhibited the typical morphology described in *C*. *intestinalis* [18,28,29]. Moreover, papillae of both types showed the same neuronal arrangements (in terms of number and location) previously described in *C*. *intestinalis* [25,30,31]. The lack of differences in the features of the papillae suggests that type A and type B larvae have a similar mechanism of substrate selection and attachment.

### Larval morphology and ascidian taxonomy

Ascidian taxonomy is mainly based on adult morphology (but see also [32]), however in some cases larvae are of great help, or even indispensable, to discriminate species. Larval morphology is especially relevant in ovoviviparous and viviparous ascidians with complex larvae, thus mainly in species of the order Aplousobranchiata and in Stolidobranchiata of the subfamily Botryllinae.

In Aplousobranchiata the larvae are of great importance since they are brooded within the parental body and reach maturity after a relatively long period of gestation [33]. Classic examples of the larval importance in Aplousobranchiata can be found in the genus *Didemnum*. This is the case, e.g., of *Didemnum maculosum* that differs from *Didemnum candidum* only in the larval morphology (in particular in the number of anterior papillae) [34] [35]. Another similar case is that of *Didemnum albidum* [36] and *Didemnum romssae* [37].

In Stolidobranchiata, only colonial species belonging to the subfamily Botryllinae produce complex larvae whose features can be essential for species discrimination. For example, the larvae of *Botrylloides violaceus* possess up to 30 anterior blood ampullae, whereas those of the other Botryllinae species exhibit only eight ampullae [38]. The larva of *B. violaceus* bears therefore the main taxonomic character that distinguishes this species from its closest relatives.

The order Phlebobranchiata, in which *C*. *intestinalis* is commonly included (but see also [39,40,41]), consists of oviparous species producing many small eggs that develop into simple larvae. Although in the *Ciona* genus the species discrimination is based mainly on the adult anatomy [42], other morphological elements have been also taken into account, such as egg size [43] and follicle cell morphology [44]. To our knowledge, the larval morphology has never been used before to discriminate species within Phlebobranchiata. However, our morphometric and histological analyses of *C*. *intestinalis* larvae clearly indicate that type A and type B larvae can be discriminated on the basis of the “trunk shape” character (Figs 3 and 4).

## Conclusions

Our data confirm that the taxonomy of the species *C*. *intestinalis* needs to be revised and demonstrate the possibility to distinguish *C*. *intestinalis* types A from type B on the basis of qualitative and quantitative morphological characters of the larvae. Indeed, type B is clearly identified by having a longer pre-oral lobe, longer and relatively narrower total body length, and shorter ocellus-tail distance than type A. The cross-validated discriminant functions were able to correctly classify 93 to 95% of the larvae utilizing a maximum of four morphometric parameters. We also demonstrated that these discriminant functions work well for the classification of larvae coming from additional populations, other than those analyzed here, and at stages not exactly corresponding to that of late-swimming larvae investigated here. Most interestingly, the differences between type A and type B larvae are particularly strong when comparing samples obtained from adults of Plymouth, *i.e.*, the region of sympatry, invoking the possibility of ecological character displacement.

The discriminating factor “trunk shape” of late larvae here identified adds to another morphological discriminating character regarding the adult tunic that we found in a parallel study on the morphology of the adults [16]. The discriminating character of the adult refers to the presence, exclusively and in all type A individuals, of tubercular prominences that correspond to those described and figured by Hoshino & Tokioka [45] in the Japanese species *Ciona robusta*. Therefore, we conclude that type A and type B are two distinct species, morphologically distinguishable based on both larval and adult characters. As presented and widely discussed in [16], in future studies the scientific community should refer to *C*. *intestinalis* type A as *C. robusta* Hoshino & Tokioka 1967 and to *C*. *intestinalis* type B as *C*. *intestinalis* Linnaeus 1767.

## Material and Methods

### Animals and crosses

Adults of *C*. *intestinalis* were sampled in four European localities from 2011 to 2013 (Table 1). No specific permits were required for the described field studies because the sampling locations are not privately owned or protected in any way. The field studies did not involve endangered or protected species.

To ascertain the type of individual sampled in the sympatric area of Plymouth, molecular analyses were also performed (see below). Gametes collected from the gonoducts were used for cross fertilization between individuals of the same type. Embryos were let to develop at 18°C in a thermostatic chamber, thus type A and type B larvae were reared in the laboratory under the same conditions. Larvae at hatching (early swimming larva, St. 26 according to FABA2 - http://chordate.bpni.bio.keio.ac.jp/faba2/2.2/top.html, St. 0 according to Chiba [19]), 4 h after hatching (mid swimming larva, St. 28 according to FABA2, St. 1 according to Chiba) and 6 h after hatching (late swimming larva, St.29 according to FABA2, St. 2 according to Chiba) were fixed for 1 h in 4% paraformaldehyde at room temperature. The total number of analyzed crosses and larvae is reported in Table 1.

### Morphometric analysis of the larvae

Fixed larvae, obtained by *in vitro* fertilization, were rinsed in 0.1 M PBS, mounted in 80% glycerol on glass slides and photographed with a Leica digital camera mounted on an optical microscope. Images were analyzed by Adobe Photoshop CS3. For each larva, we measured the following five parameters, also described in Fig 1:

1. length of the tail;
2. length of the trunk;
3. maximum height of the trunk;
4. length of the pre-oral lobe;
5. distance between the ocellus and the insertion point of the tail.

Only in few cases it was difficult to precisely measure the length of the pre-oral lobe and the ocellus-tail distance (see Table 1).

We used linear mixed models to test morphological differences between type A and type B larvae. Mixed models allow testing differences between groups, while taking into account complex clustering structures. In our analysis, we considered the five morphological parameters as dependent variables and species identity as independent variable. Population and clutch identity were considered as random factors. Mixed models were run using the package nlme in R [46].

We used Discriminant Analysis (DA) to ascertain whether morphological data can be used to unequivocally ascertain species identity on the basis of morphological features. DA is a multivariate analysis, that can be used to assess to what extent a set of quantitative explanatory variables (*e*.*g*. morphological variables) can allow to classify items in qualitative groups (the response variable, *e*.*g*. species identity). After the identification of significant differences among groups, DA proceeds to find the linear combinations of predictors that best discriminate among the groups, and test the discriminatory ability [47]. In DA, we used the five morphological parameters as explanatory variables, and species identity as the qualitative groups. Morphological parameters are strongly correlated among them. We therefore used a backward stepwise approach to identify the smallest set of parameters allowing discrimination. In turn, the least significant factor was removed from the model, and the DA was re-run using the remaining factors, until all the factors showed a significant contribution to the DA. Furthermore, it may be useful performing species identification from one single variable, even from pictures without a bar scale (*i*.*e*. without unit of measurement). We therefore performed a DA using the ratio between pre-oral length and trunk height (PL/TH ratio) as the only predictor.

The discriminatory ability of DA was assessed using cross-validation. In turn, each case (test case) was removed from the dataset, and a DA function was developed using the remaining cases. This DA was then used to classify the test case, and we then assessed whether the DA correctly allowed classifying the test case. This procedure was repeated using each larva. Our data have a strong nested structure (each species includes multiple populations, and each population includes multiple clutches). We therefore used 300 bootstraps, stratified by population and by clutch, to calculate 95% CI of the coefficients of the discriminant function, and to assess the discriminatory ability of DA. DA was performed using SPSS 19.0 (2010 SPSS Inc. and IBM Company).

### Light and transmission electron microscopy

For histology of larvae, early, mid and late larvae were fixed in 1.5% glutaraldehyde buffered with 0.2 M sodium cacodylate, pH 7.4, plus 1.6% NaCl. After washing in buffer and postfixation in 1% OsO_4_ in 0.2 M cacodylate buffer, the specimens were dehydrated and embedded in Araldite. Sections (1 μm) were counterstained with Toluidine blue. Transverse, frontal and sagittal serial sections of larvae were cut. All photos typeset in Corel Draw X3.

For 3D reconstructions, late-larvae of types A and B were serially cross sectioned (1 μm); sections were stained with toluidine blue. Light microscopic images were recorded with a digital camera (Leica DFC 480) mounted on a Leica DMR compound microscope. Images were aligned using Adobe Photoshop CS on a Windows 7 computer. Based on the resulting stack of images, 3D models of the anatomy of all organ systems were created in Amira 5.3.3 software (Mercury Computer Systems, Berlin).

### Immunolabeling of nervous fibers

To count the number of peripheral neurons in the trunk and in the tail, we immunostained nervous fibers of whole mount larvae with an anti-acetylated tubulin antibody. Briefly, early, mid and late swimming larvae obtained from adults of Roscoff (type B) and Venice (type A) were fixed in 4% paraformaldehyde, washed several times in 0.1 M PBS, treated for 20 minutes with 0.1% Tween and 0.25% di Triton in PBS to permeabilize cellular membranes. Then, they were incubated over night at 4°C with a mouse anti acetylated tubulin antibody (Sigma, Italy) diluted 1/200 in PBS. After several washes in PBS, the samples were incubated with the secondary antibody Alexa Fluor anti mouse diluted 1/400 overnight at 4°C. The sample were extensively rinsed and then mounted on glass slide with DABCO (1,4 diazabicyclo [2,2,2] octane) and observed with a confocal laser microscope (Leica TCS-NT, Leica Microsystems, Heidelberg, Germany). Images were processed with Photoshop CS3 software.

### Molecular analyses

Molecular analyses were carried out only on adults and larvae coming from the region of sympatry, *i.e.*, from Plymouth. Parent genotyping using restriction enzymes was performed using one mitochondrial (*cox1*) and three nuclear markers (*vAChTP*, *CiCesA*, and *patched*) as described in Sato et al. [14]. Genotyping of adults and the relative larvae of the F1 progeny based on two mitochondrial (mt) markers were carried out according to [12]. Details of the molecular analyses and the laboratory crosses between adults from Plymouth, including few hetero-type crosses (*i.e.*, type A x type B and type B x type A), are reported in S5 File.

Briefly, total DNA was extracted from specimens preserved in 99% ethanol using the CTAB method. For each cross, the extraction was performed from the ovary of parental animals and from a small batch of F1 progeny larvae.

The used mitochondrial screening tests are based on the existence of structural differences between the mitochondrial genome (mtDNA) of type A and type B, and consist in PCR reactions and sequencing of the obtained amplicons. The tests, named mt:trnC-test and mt:NCR-test, are here detailed.

1. mt:trnC-test: amplification of the 1 kb region between *nad4* and *cox1* (primers: tn4F/tx1R), and sequencing of the amplicon portion including also the *trnF*, *atp8* and *trnC* genes (about 700 bp; sequencing primer: tg1R). This test is able to define the exact genomic position of *trnC*, which is translocated in the mtDNA of type A compared to type B.
2. mt:NCR-test: amplification and sequencing of the region between *cox3* and *nad1*, including the *trnK* gene, and characterized by the presence of a non-coding region (NCR) of 85 nt only in type A but not in type B. Therefore, the amplified fragment will be about 700 bp in type A, and 600 bp in type B. The amplicon is obtained using the primer pair tx3F/tn1R, and the sequencing (although not strictly necessary) is carried out with the tx3F primer.

All PCRs were performed with the Expand High Fidelity PCR System (Roche) or Phusion High-Fidelity DNA polymerase (Finnenzyme) following the PCR conditions and using the primers described in [12].

Both mitochondrial markers (mt:trnC and mt:NCR) confirm the type A and type B assignment determined on the basis of adult morphological characters described in [14].

As reported in S5 File, larvae of the F1 generation have exactly the same sequence of the mother (*i.e.*, the adult from which the egg was taken) even in the few analyzed hetero-type crosses. In crosses between adults of the same type (both A or both B), the few nucleotide differences found between parental individuals testify the strictly maternal inheritance of the mtDNA (S5 File). On the overall, the mt differences observed between parental individuals of the same type consist in:

- only 1 substitution over 1164 analyzed positions in type A;
- a maximum of 2 substitutions plus 1–4 indels over 1083 analyzed positions in type B.

The identified differences are mainly synonymous substitution in the 3rd codon position of the atp8 gene, while one non-synonymous substitution is present in *nad1* of type B. Finally, no mutations have been observed in the F1 progeny compared to the maternal mtDNA.

These data demonstrate the maternal inheritance of the mitochondrial DNA in homo- and hetero-type crosses between Plymouth sympatric individuals. We did not identify cases of “paternal leakage”, *i.e.*, transmission of paternal mtDNA to the progeny, a phenomenon often associated to inter-species crosses. However, our tests were not based on quantitative PCR so they could be unable to detect small amounts of paternal mtDNA diluted in a high quantity of maternally inherited mtDNA. In order to define the capability of the NCR-test to detect the coexistence of the two different mtDNA types in the same sample, we have performed some NCR-tests on artificial samples created mixing in different ratios the total DNA of a type A (168) and a type B (165) adult. The analyzed ratios are: 50:50; 70:30; 90:10 and 99:1. Moreover, for each ratio, we have made and tested the two alternative combinations (*i.e.*, 70typeA: 30typeB and 30typeA: 70typeB). The results show that both the fragments typical of type A (700 bp) and type B (600 bp) are amplified (and easily visible on an agarose gel) starting from a mix with up to a 90:10 ratio between type A and type B. On the contrary, only the most abundant mtDNA is detected in a mix containing a 99:1 ratio between type A and type B. We can conclude that in the analyzed hetero-type crosses (A x B or B xA) there is no paternal leakage with paternal mtDNA ≥ 10%, while we cannot exclude the presence of paternal mtDNA in a percentage ≤1%.

## Supporting Information

S1 File The two discriminant equations calculated on four and on a single larval variable. The discriminant equation “4v” was calculated on the four larval variables strictly related to the trunk, excluding the tail length. The discriminant equation “1v” was calculated on the single variable PL/TH ratio (ratio between the pre-oral lobe length and the trunk height).
(DOC)

S2 File Data on photos of larvae of *C*. *intestinalis*, selected from published papers and evaluated in this study through morphometric analyses. PL/TH ratio = ratio between pre-oral lobe length and trunk height; DA-4v: discriminant analysis based on four variables strictly related to the trunk; DA-1v: discriminant analysis based on one variables, that is the only PL/TH ratio. The “type” column indicates *C*. *intestinalis* larval classification inferred from the sampling locality reported in the publication.
(XLS)

S3 File 3D animation of a whole larva of type A (A-L) (the correct visualization of the 3D-PDF requires Acrobat Reader version 7.0 or higher). See the legend of Fig 7 in the main text for symbols and colour meaning.
(PDF)

S4 File 3D animation of a whole larva of type B (A’-L’) (the correct visualization of the 3D-PDF requires Acrobat Reader version 7.0 or higher). See the legend of Fig 7 in the main text for symbols and colour meaning.
(PDF)

S5 File Molecular characterization of larvae obtained from laboratory crosses of adults sampled in Plymouth (the region of sympatry of *C*. *intestinalis* type A and type B). (DOC)
