# Supplementary material for: Morphological Differences between Larvae of the Ciona intestinalis Species Complex: Hints for a Valid Taxonomic Definition of Distinct Species
Source: PLoS One. 2015 May 8;10(5):e0122879. doi: 10.1371/journal.pone.0122879 (PMC4425531; doi:10.1371/journal.pone.0122879)
Supplement: S1 File — The discriminant equation “4v” was calculated on the four larval variables strictly related to the trunk, excluding the tail length. The discriminant equation “1v” was calculated on the single variable PL/TH ratio (ratio between the pre-oral lobe length and the trunk height). (DOC) [file pone.0122879.s001.doc]

**DISCRIMINANT FUNCTIONS to classify *C. INTESTINALIS* larvae**

***Discriminant function DA-4v: classification using four morphological variables***

Individuals with D scores > 0.5 are classified as type B, while individuals with D scores < 0.5 are classified as type A

***Discriminant function DA-1v: classification using only one morphological variable***

Individuals with D scores > 0.346 are classified as type B, while individuals with D scores < 0. 346 are classified as type A
