# Supplementary figures and images for: Morphological Differences between Larvae of the Ciona intestinalis Species Complex: Hints for a Valid Taxonomic Definition of Distinct Species
Source: PLoS One. 2015 May 8;10(5):e0122879. doi: 10.1371/journal.pone.0122879 (PMC4425531; doi:10.1371/journal.pone.0122879)

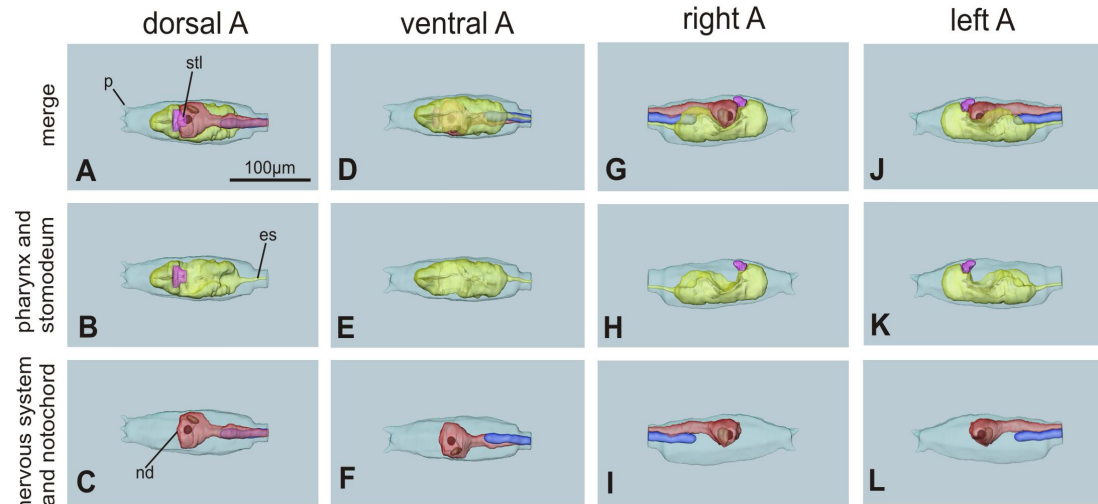

Supplement: S3 File — See the legend of Fig 7 in the main text for symbols and colour meaning. (PDF) [file pone.0122879.s003.pdf]

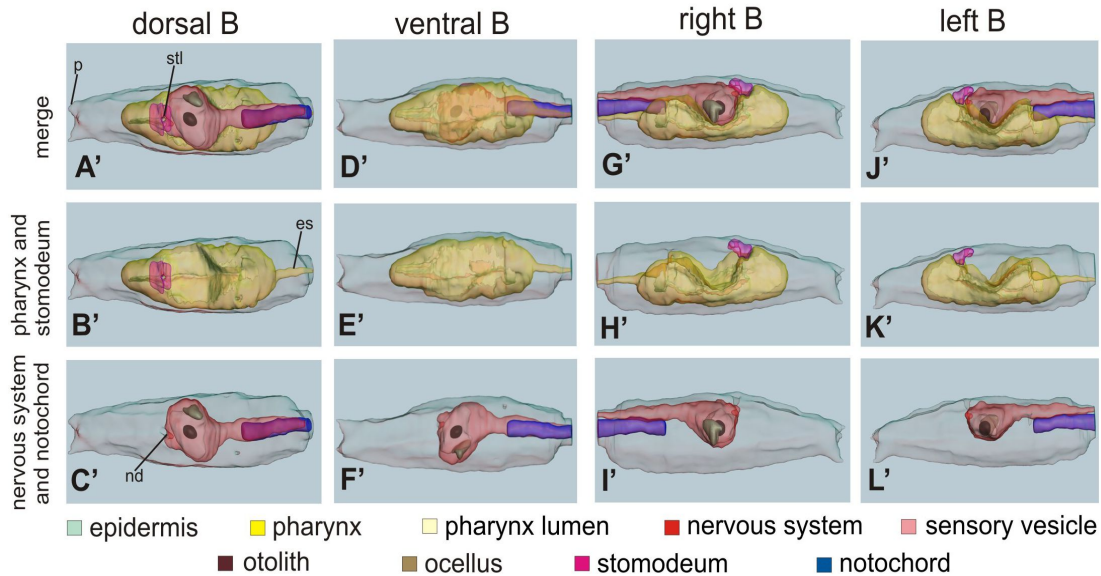

Supplement: S4 File — See the legend of Fig 7 in the main text for symbols and colour meaning. (PDF) [file pone.0122879.s004.pdf]
