# Supplementary material for: Morphological Differences between Larvae of the Ciona intestinalis Species Complex: Hints for a Valid Taxonomic Definition of Distinct Species
Source: PLoS One. 2015 May 8;10(5):e0122879. doi: 10.1371/journal.pone.0122879 (PMC4425531; doi:10.1371/journal.pone.0122879)
Supplement: S5 File — (DOC) [file pone.0122879.s005.doc]

**Table 1**. Adults (1A) and F1 progeny (1B) of *C. intestinalis* specimens from Plymouth investigated in this study through molecular analyses. Laboratory hybrids are reported in red or underlined. P: parental

Table 1A

| **Adult name**  **(from wild)** | **Morphological classification according to Sato et al (2012)** |
| --- | --- |
| 162 | natural hybrid |
| 163 | natural hybrid |
| 164 | natural hybrid |
| 165 | Type B |
| 166 | Type B |
| 167 | Type B |
| 168 | Type A |
| 169 | Type A |

**Table 1B**

| **F1 progeny name (larvae)** | **P egg** | **P sperm** | **Cross**  **(egg x sperm, based on molecular analyses)** |
| --- | --- | --- | --- |
| 5 | 165 | 166 | BxB |
| 6 | 166 | 167 | BxB |
| 8 | 168 | 169 | AxA |
| 9 | 169 | 168 | AxA |
| 10 | 165 | 168 | BxA |
| 11 | 166 | 169 | BxA |
| 12 | 168 | 165 | AxB |
| 13 | 169 | 166 | AxB |
| 14 | 167 | 168 | BxA |

**Table 2.** Assignment of adult specimens sampled in Plymouth (UK) based on morphological characters and molecular markers. NH: natural hybrid according to Sato et al (2012); SEQ: sequencing of the amplified fragment; RE: restriction analysis of the amplified fragment; ND: not determined; A: *C. intestinalis* type A; B: *C. intestinalis* type B

| **Adult name** | **Morphological classification according to Sato et al (2012)** | **mt:trnC** | **mt:NCR** | **mt:COI** | **vAChTP** | **CiCesA** | **Patched** |
| --- | --- | --- | --- | --- | --- | --- | --- |
|  |  | **SEQ** | **SEQ** | **RE** | **RE** | **RE** | **RE** |
| 162 | NH | A | A | A | A | A | A |
| 163 | NH | A | A | A | A | A | A |
| 164 | NH | A | A | A | A | A | A |
| 165 | B | B | B | B | B | B | B |
| 166 | B | B | B | B | B | B | B |
| 167 | B | B | B | B | B | B | B |
| 168 | A | A | A | A | A | A | A |
| 169 | A | A | A | A | A | ND | A |

**Table 3.** Assignment of larvae from Plymouth obtained from laboratory crosses based on two mitochondrial markers. Laboratory hybrids are reported in red or underlined. SEQ: sequencing of the amplified fragment: A: *C. intestinalis* type A; B: *C. intestinalis* type B

| **Parent (adult name)** | | **Cross**  **(egg x sperm)** | **F1 progeny name (larvae)** | **mt:trnC (SEQ)** | **mt:NCR (SEQ)** |
| --- | --- | --- | --- | --- | --- |
| **P egg** | **P sperm** |  |  |  |  |
| 165 | 166 | BxB | 5 | B | B |
| 166 | 167 | BxB | 6 | B | B |
| 168 | 169 | AxA | 8 | A | A |
| 169 | 168 | AxA | 9 | A | A |
| 165 | 168 | BxA | 10 | B | B |
| 166 | 169 | BxA | 11 | B | B |
| 168 | 165 | AxB | 12 | A | A |
| 169 | 166 | AxB | 13 | A | A |
| 167 | 168 | BxA | 14 | B | B |

**Table 4A.** Nucleotide differences observed between type A adults and between *in vitro* hybrids produced by type A eggs x type B sperm hetero-crosses. NH: natural hybrids according to Sato et al (2012). Crosses that gave rise to larvae are indicated as “Egg Parent type x Sperm Parent type”. =: absence of nucleotide differences. The gene containing the difference is reported, together with the affected codon. Laboratory hybrids are underlined.

| **Type** | **Sample (adult or F1 progeny name)** | **mt:trnC (931 sites)** | **mt:NCR**  **(630 sites)** |
| --- | --- | --- | --- |
|  |  | **atp8:Val (Syn)** |  |
| A (NH) | 162 | C | = |
| A (NH) | 163 | C | = |
| A (NH) | 164 | C | = |
| A | 168 | C | = |
| A | 169 | G | = |
| 168A x 169A | 8 | C | = |
| 169A x 168A | 9 | G | = |
| 168A x 165B | 12 | C | = |
| 169A x 166B | 13 | G | = |

**Table 4B.** Nucleotide differences and indels observed between type B adults and between *in vitro* hybrids produced by type B eggs x type A sperm hetero-crosses. Crosses that gave rise to larvae are indicated as “Egg Parent type x Sperm Parent type”. The genes containing nucleotide difference are shown together with the affected codon or the consequent amino acid substitution. Differences affecting a non-coding region are indicated by the name of the flanking genes, separated by a “/”. Differences affecting a pattern partially covering two genes are indicated by the name of the genes, separated by a “+”. Laboratory hybrids are underlined.

| **Type** | **Sample (adult or F1 progeny name)** | **mt:trnC (931 sites)** | | | | | **mt:NCR**  **(548 sites)** |
| --- | --- | --- | --- | --- | --- | --- | --- |
|  |  | **atp8:Leu (Syn)** | **trnF/atp8** | **atp8/trnG(AGR)** | **trnL(CUN)+**  **trnS(UCN) ^(a)^** | **cox1:Gly**  **(Syn)** | **nad1: Thr->Ile (NonSyn)** |
| B | 165 | A | 7A | AAT | 12A | A | C |
| B | 166 | G | 8A | AAT | 15A | G | T |
| B | 167 | G | 8A | AATAAT | 16A | G | T |
| 165B x 166B | 5 | A | 7A | AAT | 12A | A | C |
| 166B x 167B | 6 | G | 8A | AAT | 15A | G | T |
| 167B x 168A | 14 | G | 8A | AATAAT | 16A | G | T |
| 165B x 168A | 10 | A | 7A | AAT | 12A | A | C |
| 166B x 169A | 11 | G | 8A | AAT | 15A | G | T |

(a): The length of the omolpolymeric “A” stretch, covering the end of trnL(CUN) and the beginning of trnS(UCN) (position 11096 of the AM292218 complete mtDNA sequence of type B), cannot be reliably determined because of low sequence quality. The table reports the minimum length of the omopolymer

**REFERENCES**

Sato A, Satoh N, Bishop JDD (2012) Field identification of 'types' A and B of the ascidian Ciona intestinalis in a region of sympatry. Marine Biology 159: 1611-1619.
